# Stingless bees: uses and management by meliponiculturist women in the Chaco region of Bolivia

**DOI:** 10.1186/s13002-022-00574-0

**Published:** 2023-01-10

**Authors:** Marcia Adler, Luciana Escóbar-Márquez, Maria Teresa Solis-Soto, Carlos F. Pinto

**Affiliations:** 1 Universidad Mayor Real Y Pontificia de San Francisco Xavier de Chuquisaca, Calle Estudiantes 56, 0000 Sucre, Bolivia; 2 Center for International Health, University Hospital Munich (LMU), Ziemssenstr. 1, 80336 MunichMunich, Germany

**Keywords:** Ethnobiology, Meliponini, Traditional knowledge, Medicinal honey, Conservation, Relative cultural importance

## Abstract

**Background:**

Stingless bees have a great value as main pollinators of wild flowering and cultivated plants, thus playing a fundamental role in the maintenance of biodiversity and food security in Latin America. Despite their importance, stingless bees face numerous threats causing alarming population declines. Moreover, stingless bees have a great cultural and traditional value, since most products from the hive are used for a wide variety of purposes. A growing number of initiatives are encouraging the breeding of these bees, through training courses and modern management techniques. This study documents the knowledge on stingless bees and their products that meliponiculturists from the Chaco region of Bolivia have, as well as the influence that meliponiculture initiatives have on the management and general knowledge of the bees.

**Methods:**

Local richness and diversity of stingless bees was calculated using Hill numbers. Structured interviews were conducted with 59 meliponiculturists in order to characterize traditional and formal knowledge on stingless bees and meliponiculture. Generalized linear models were applied to assess the influence of training courses on the management of the bees. Also, a relative cultural importance index was calculated for each species.

**Results:**

Twelve Meliponini species were identified, and 15 local names were reported with morphological, defensiveness behavior, and nest description. There was no significant difference in the knowledge between different ethnical backgrounds or ages. A significant difference was observed in the use of supplementary feeding and assisted division, but none in the success in racking hives or in pest management, regarding the number or courses taken. The relative cultural importance index recorded 30 specific uses for bee products grouped in four categories, from which 29 were attributed (but not exclusively) to *Tetragonisca angustula*, making it the most versatile species.

**Conclusions:**

The products of the hive, especially honey, are used for a wide variety of purposes, mostly in medicine. These uses are mostly attributed to just one species, *T. angustula*, in coincidence with what was taught in meliponiculture training courses by NGOs. The influence of formal knowledge is mostly positive, but it is recommended that other meliponini species are taken into account as well.

**Supplementary Information:**

The online version contains supplementary material available at 10.1186/s13002-022-00574-0.

## Background

The importance of bees as the main pollinator of a great variety of wild flowering plants and almost 70% of all cultivated plants is worldwide recognized [1–3], but most people are unaware that more than 20,000 species of bees around the world are responsible for this ecosystemic service [4]. In the Neotropics, around 10% of the approximately 5,000 bee species described belong to the tribe Meliponini, commonly known as stingless bees on account of one of their main characteristics: a stunted sting [5–7]. Depending on the species, these eusocial bees normally live in permanent colonies with one queen and between 3000 and 100,000 workers [8]. Stingless bees are the most common and abundant bees in many areas of this region and represent approximately 70% of all eusocial bees [8, 9]. Hence, they play a fundamental role in the maintenance and perpetuity of biodiversity in many ecosystems; moreover, they are crucial for food security in Latin America as they are important pollinators of a vast number of cropping systems [8, 10, 11].

Despite their importance, bees and many other pollinators face numerous threats, including habitat loss due to deforestation, wildfires, changes in land use, excessive use of pesticides, and climate change, which are causing alarming population declines that will have severe consequences for the ecosystems and global human diet, health, and economies [2, 3, 12, 13]. For these reasons, efficient and long-term strategies for conservation of stingless bees (and other pollinators) is a pressing matter [14, 15]. Furthermore, there is a huge knowledge gap on these bees, since only about 6% of scientific publications deal with stingless bees compared to other eusocial bees such as honeybees and bumble bees [9].

Stingless bees also have a great cultural value for many indigenous and rural populations [16–19]. The breeding of stingless bees is known as meliponiculture and dates back to the pre-Columbian Maya civilization in Mesoamerica, as documented through ancient paintings [20–22]. These bees are bred mainly for their honey, which they stock in cerumen pots, making it relatively easy to harvest [23], and for other important resources obtained from the hives such as cerumen, propolis, and pollen [18, 24]. Hive products are used for a wide variety of religious, medical, nutritional, and economic purposes [16, 25, 26]. In Bolivia for example, the Yuqui, a Tupi–Guaraní-speaking folk from the Chapare region, use a mixture of cerumen and plant resin to manufacture arrows for hunting [27]; the Siriono, an indigenous population from the Amazonas, feed on larvae (actually the complete brood cells) and pollen, and use honey to prepare fermented drinks [28]; and the Ayoreos of Southern Bolivia have a particularly strong connection to bees and hive derivatives, consuming honey as a sweet and for religious purposes, and cerumen as a key material to make anything from instruments to cloths [16]. Likewise, many rural populations benefit from stingless bee products like honey, pollen, or propolis, either for self-consume as medicine to treat various diseases or as an additional income source by selling these products [20]. While meliponiculture is widely spread in many regions of Latin-American, in Bolivia meliponiculture is not very common; instead, bee products are most commonly harvested from wild hives, thus destroying them and endangering bee populations [16].

Despite their cultural importance, traditional uses of honey and other hive products have decreased over the last decades since it has become harder to find wild hives near villages due to over-harvesting and logging, which reduce their habitat and suitable nesting sites [29]. Hence, many people are starting to practice apiculture instead of meliponiculture because honeybees produce more honey [30]. On the other hand, in Bolivia and many other Latin-American countries, there has been a great number of government, NGOs, and private initiatives to enhance the development of meliponiculture in rural areas. For example, in Brazil and Colombia meliponiculture has developed very quickly, triplicating the number of bee boxes in 2005 and becoming important for the economy of many rural and Indigenous families [20]. This fast development is due to the advances in the knowledge and understanding of meliponine biology, which gave way to improvement in management techniques, especially in the division of the hive, processing of honey, and colony food supplementation, as well as to social and governmental projects which encourage meliponiculture and these new techniques [20, 31]. In Bolivia, government and NGOs’ social initiatives to promote meliponiculture are becoming increasingly popular especially among women in rural areas as a source of sustainable incomes and also as a way of empowerment. Although most of these initiatives tend to leave behind cultural aspects and are mainly focused on sustainably improving the economic situation of rural communities, by simply promoting meliponiculture they are also helping bees’ conservation [32].

In the Boliviano-Tucumano forest of Chuquisaca in southern Bolivia, in the municipalities of Villa Vaca Guzmán and Monteagudo, a large-scale meliponiculture initiative from a local Foundation (PASOS) has taken place since 2015, where more than 200 women from different communities and ethnical backgrounds have been trained in meliponiculture. PASOS is financed by the German Agency for International Cooperation GmbH (GIZ for their initials in German—Deutsche Gesellschaft für Internationale Zusammenarbeit GmbH) and aims to increase women's own income in the Chaco region of Chuquisaca through the ecological business development of the production of stingless bees’ honey. To train such a large number of producers, PASOS focuses on training representatives from each community who later replicate what they learn with the other women of the communities. Additionally, communal meliponaries (also called field schools) have been installed in some regions so that practical training can be carried out locally.

In the Chaco region in Chuquisaca, the main economic activities are agriculture and raising livestock. Most of the products are used for self-consumption, although some are also commercialized. The main products are peanuts, chili peppers, corn, beans, and citrus fruits; meliponiculture and apiculture have also become important, leading to small-scale production consumed mainly by the producing families. The Villa Vaca Guzmán and Monteagudo municipalitys are multiethnic, mainly from Guarani, Quechua, and Spanish stock [33, 34]. Both municipalities reported a high percentage of the population living in poverty due to Unsatisfied Basic Needs criteria (Monteagudo 74.4%, Villa Vaca Guzmán 82.1%), higher than the national average (70.9%) [35].

In order to improve conservation and management strategies in the Chaco region, it is important to document the current state of knowledge that women have about stingless bees’ management and meliponiculture practices. In this study, the resulting knowledge from training activities is referred to as formal knowledge, while previous knowledge, even though it is not ancestral, is referred to as traditional. The present study documents, characterizes, and analyzes structured interviews of women from rural communities in the Chaco-Chuquisaqueño region, in order to better understand traditional and formal knowledge of stingless bees and practices in meliponiculture performed by them. It is also documented how traditional practices have changed or evolved after having formal education on meliponiculture through NGOs acting in the region. Additionally, bee surveys and identification were conducted to assess stingless bee diversity of the region, and thus explore its relationship with cultural importance and to evaluate its conservation status.

## Methods

### Study area

**Fig. 1:**
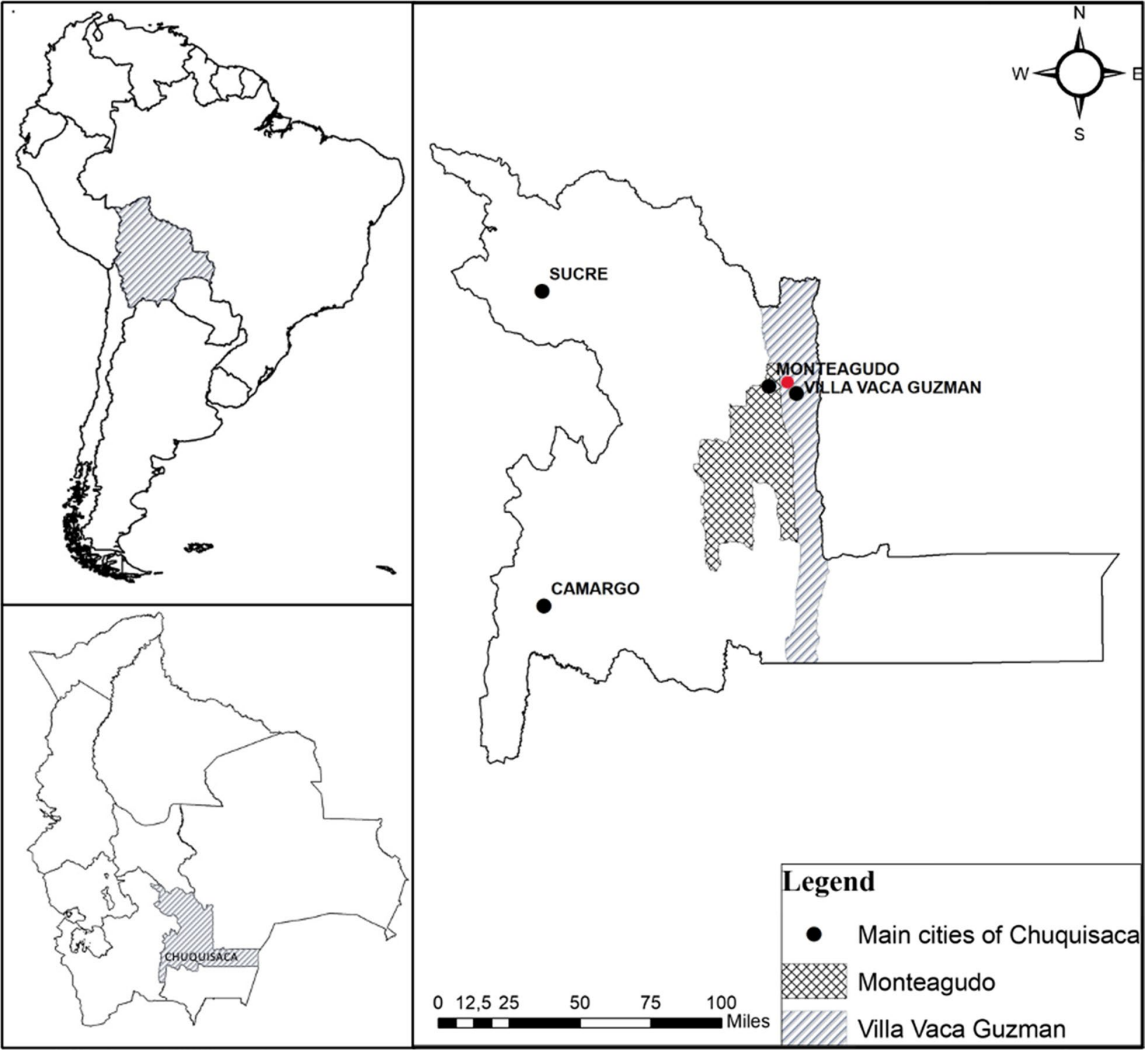
Map of study area. The red dot indicates where the bee surveys were taken, within the communities of Ticucha and La Tapera, at Villa Vaca Guzmán municipality in Chuquisaca–Bolivia

The study was carried out in the municipalities of Villa Vaca Guzmán, best known as Muyupampa (19°54′ S, 63° 43′ W, 600–2800 m.a.s.l.) and of Monteagudo (19° 30′ S, 63° 45′ W, 2000 m.a.s.l.), both located in the southeastern region of the Chuquisaca department, Bolivia. The northern region of both municipalities, where the study took place, belongs to the biogeographic region of the Boliviano-Tucumano forest, characterized by a mesotropical subhumid pluviseasonal bioclimate. This region presents high montane ecological floors with an altitude of 2700–3800 m above sea level [36, 37], where some of the main tree species are “palo Barroso” *(Blepharocalyx salicifolius)*, “mato” *(Myrcianthes pseudomato)*, “laurel” *(Cinnamonum porphyria)*, “cedro” *(Cedrela lilloi)*, “pino negro” *(Podocarpus parlatorei)*, “yuruma” *(Myrsine coriaceae)*, among others [38]*.* In general terms, this area is well conserved, since a large proportion of it lies within the limits of the National Park “Serranía del Iñao” (Fig. 1).

### Stingless bee survey and identification

Bee surveys to determine local bee diversity were conducted during the end of the 2022 rainy season (February 11–16, March 16–24, and May 6–8) in the surroundings of the communities of La Tapera (19° 40′ 42.75′′ S, 63° 48′ 58.4′′ W) and Ticucha (19° 37′ 23.01′′ S, 63° 49′ 29.9′′ W), approximately 20 km northeast from Monteagudo. Seven transects (200 m long) were established at a range of no further than 2 km from the communities (4 in La Tapera, 2 in Ticucha and 1 in La Angostura). Sites were chosen based on high accessibility and low human traffic (Fig. 1). A group of three pan traps of different colors (white, blue, and yellow) were placed every 20 m along the transects, so that every transect had 33 pan traps in total [39]. Although previous studies had shown that the minimum distance between color traps should be 5 m [40], it was decided to place them every 20 m to cover a larger area. Also, three air white traps were hung approx. 3 m above ground along the same transects, one every 100 m. All traps were filled two-thirds with soapy water (to break the surface tension of the water), which was prepared by mixing a few drops of unscented liquid dishwashing detergent per liter of tap water. Traps were deployed for approx. 9 h, being set up early in the morning and collected in the evening, just one time in each transect. Additionally, targeted netting surveys were conducted along the transects and around the communities. Netting time varied between 1 and 3 h, during 7 says (when de transects were set) and included actively catching and searching for stingless bees between flower patches and flowering trees.

Samples collected from the traps were placed in plastic vials containing 70% ethyl alcohol. Samples collected with the net were transferred to a lethal chamber with ethyl acetate and conserved dry before being transported to the laboratory. In the laboratory, insects were pinned, labeled, boxed, and identified to genus level with the help of specialized identification keys [41–46]. Voucher specimens were stored at the Chemical Ecology Laboratory insect collection at Universidad San Francisco Xavier de Chuquisaca, Sucre, Bolivia. Additionally, samples from some of the meliponaries were taken and identified, if possible to species level.

### Demographic profile of the subjects interviewed

Among the 52 female informants, half of them were aged between 20 and 39 (young) and half between 40 and 60+ (adults). Male participants were mainly adults (6 of the 7 participants). Most informants (54) came from communities of the municipality of Villa Vaca Guzmán, mostly from La Tapera and from Ticucha (18 from each) and other seven communities; the other five belonged to four different communities from the municipality of Monteagudo. All the interviewed persons carried out meliponiculture as an alternative activity; 45 of the women said their primary occupation was housework, three had apiculture as their main activity, and the rest of the interviewees (males and females) had other occupations such as farmer, teacher, nurse, or carpenter. All the people surveyed were asked whether they felt identified with an indigenous ethnic group; the majority (42) identified themselves as “mestizo” (this also included those who did not identify themselves with any ethnic group), and only eight interviewees identified themselves as Guarani and only seven as Quechuas.

### Stingless bee local knowledge and uses

**Fig. 2:**
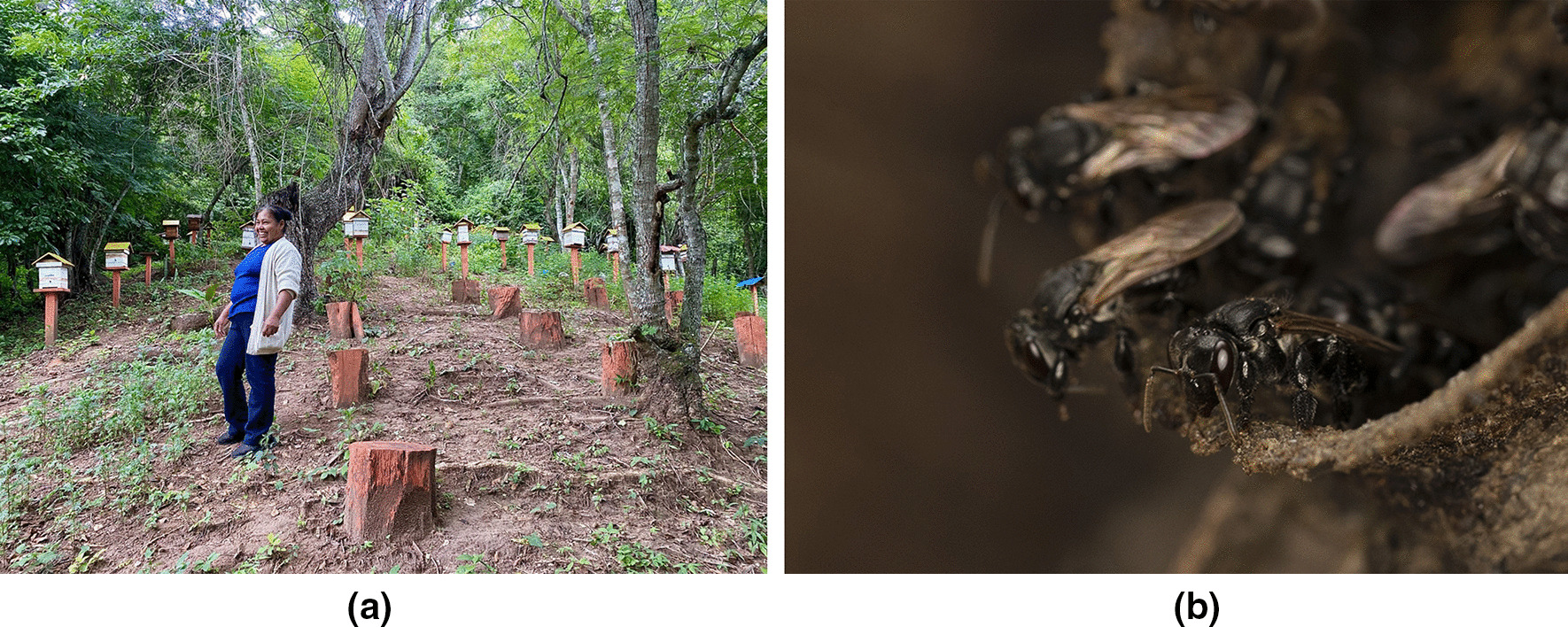
**A** One of the interviewed meliponiculturists in her meliponary. **B** *Scaptotrigona sp,* known locally as Negro

To assess and document knowledge, practice, and management techniques by local people, semi-structured interviews were conducted with 52 women and 7 men (Fig. 2). The interviewed women were part of the meliponiculture project carried out by the Foundation “PASOS.” The men interviewed were mostly these women’s husbands or relatives who were also very interested and involved in meliponiculture and had received the same training along with the women. To carry out the interviews, meetings were organized in the communities, with the participation of all meliponiculturists from Ticucha and La Tapera communities (Villa Vaca Guzmán Municipality). In Monteagudo, meliponiculture representatives from other smaller communities in the area also participated. All interested women were invited to participate in the interviews. The interviews were conducted by previously trained biology thesis students from Universidad Mayor Real y Pontificia de San Francisco Xavier de Chuquisaca (USFX), trained by the coordinators of the study. The interviews lasted approximately 1 h and were conducted in Spanish, the most spoken language in the area. On the question of ethnicity of the interviewees, “mestizo” corresponded to interviewees not belonging to indigenous ethnical group, as well as those who did not feel identified with any ethnic group in particular.

The interviews consisted of 90 questions divided into 5 parts which addressed the following topics: (a) general knowledge about stingless bees, (b) meliponiculture and hive management, (c) honey harvesting and uses, (d) other bee products and their uses, and (e) processing of hive products and their use in traditional medicine. Before the interview, the participants were informed about the study aims, and written informed consent was obtained from all those involved. The open-source app ODK collect (V. 2022.3.4) was used to carry out the interviews.

### Ethics approval and consent to participate

International ethical recommendations for research with human beings were followed to protect participants' rights and well-being. The Bioethics Committee of the Faculty of Medicine of the University of San Simón, Cochabamba, Bolivia, approved the research study protocol. An educative process was performed before the information letter. After that, a written informed consent form was provided to each participant. The questionnaire was completely anonymous, voluntary participation was always respected, the information was also handled confidentially, and any situation that could harm the communities was considered. Additionally, we adhere to the principles of the Code of Ethics of the International Society of Ethnobiology (ISE) [47] by recognizing the inalienable rights of the local community over their territories and the use of their natural resources, their right to self-determination, and the role of communities as traditional guardians of culture and ecosystems. On the other hand, the active participation of the meliponiculturists in all phases of the project was encouraged to promote capacity-building, all the communication generated in the project was socialized, and their culture and organization was respected in all steps of the project.

### Data analysis

**Fig. 3:**
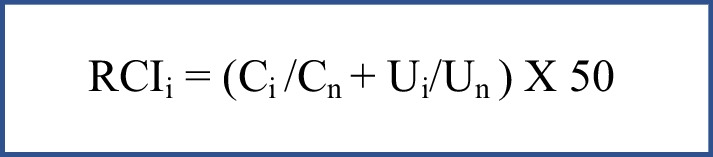
Formula used to calculate the relative cultural importance index

Local stingless bee diversity and richness were analyzed using equations based on Hill numbers *q* = 0 and *q* = 1 as proposed by Chao et al. [48]. Integrated extrapolation and rarefaction curves were developed, and thus, the estimated richness was calculated for each Hill number, where q = 0 estimates the species richness and q = 1 weighs the number of species based on relative abundance, obtaining a diversity value based on the exponential of the Shannon index. Also, a sample completeness curve was constructed to find out how complete was the sampling protocol. These analyses were carried out using the iNEXT V.2.0.20 package [48–50] from the statistics free Software R version 4.2.1. To determine the cultural importance of the different species of stingless bees, based on the information retrieved from the interviews, the relative cultural importance index (RCI) (Fig. 3) was calculated. This quantitative ethnobiological technique was developed by Bennett and Prance [51] to calculate the relative importance of medicinal plants, but an adaptation was suggested by Gonzales et al. [52] and later by Bhatta et al. [53] to analyze the cultural importance of stingless bees. For this adaptation, first main categories of use (*C*) and specific uses (*U*) for the bee products are identified (Additional file 1: Appendix 3), and the following formula used to calculate RCI.

Here, RCI_*i*_ is the relative cultural importance of species i, C_*i*_ is the number of categories reported in the interviews and C_n_ is the total number of categories, U_*i*_ is the number specific uses reported in the interviews and U_n_ is the total number of specific uses. For example, for *M. rufiventris* two of the four use categories were reported; thus, it had a *C* value of 2/4 = 0.5. Two out of the 30 specific uses were recorded; thus, it had a *U* value of 2/30 = 0.07. The combined scores were 0.57 out of a maximum possible value of 2. To express it in a scale of 100, the combined scores were multiplied by 50, thus resulting in a relative importance of 28.33 for *M. rufiventris*.

The data obtained from the interviews were systematized and ordered in frequency tables. The frequencies obtained were transformed to percentages for further analysis; moreover, generalized linear models (GLM) were used to assess whether there was any influence of the interviewees' age (young = 20–39 years old, adult = 40–60+ years old) or ethnicity (Mestizo, Guaraní and Quechua) on their traditional knowledge about bee diversity (number of reported species). Training courses were given by PASOS on the following topics: Main characteristics of stingless bees, management of modern hives, the harvest of honey and other bee products, and product transformation. GLMs were used to assess how the number of these courses (0, 1, 2, 3, 4) influenced the successful management of the bees regarding: (A) colony division (success, failure, and no division), (B) supplementary feeding (yes and no), (C) racking of wild hives into boxes (success and failure), and (D) practice of pest management (yes and no). In all cases, a quasipoisson distribution was used. A Spearman’s rank correlation was used to test for an association between the number of hives bred for each species and the calculated RCI. This was done to see whether the most bred species are also the most culturally important.

## Results

### Stingless bee survey and identification

During the field trips, a total of 36 individuals were collected using the different sampling methods. Twelve morphospecies of stingless bees were identified, which belonged to 8 genera (*Lestrimelitta, Melipona, Paratrigona, Plebeia, Scaptotrigona, Scaura, Tetragonisca,* and *Trigona*), with *Plebeia* being the most abundant species (Table 1).

**Table 1:** Number of stingless bee species (*n*) collected and identify to genera in the municipality of Muyupampa, Bolivia

Species	*n*
*Plebeia* sp	7
*Trigona* sp	5
*Trigona* sp2	4
*Melipona* sp	3
*Paratrigona* sp	3
*Scaptotrigona* sp	3
*Tetragonisca* sp	3
*Lestrimelitta* sp	2
*Scaptotrigona* sp2	2
*Scaura* sp	2
*Melipona* sp2	1
*Plebeia* sp2	1
Total	36

**Fig. 4:**
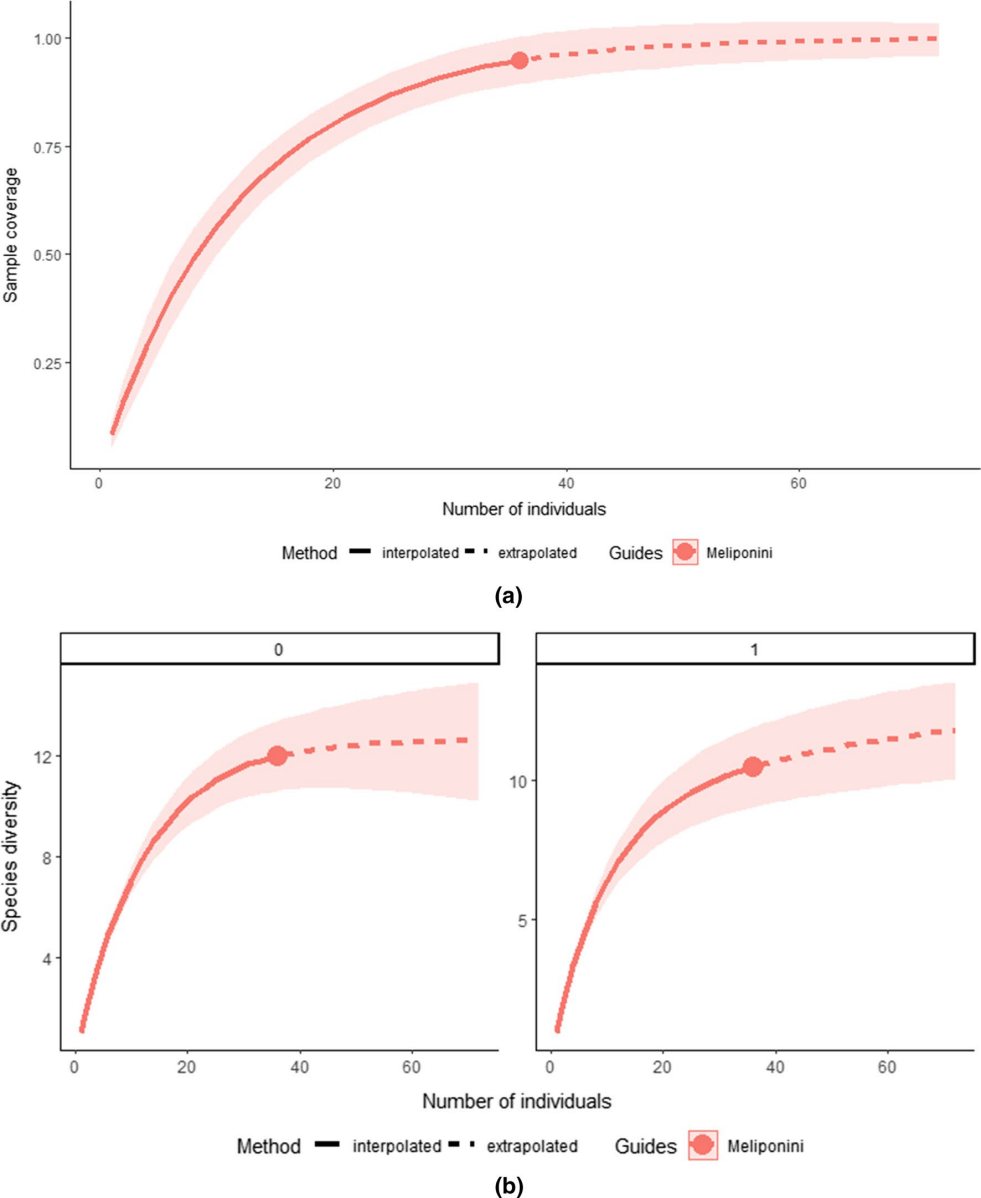
**a** Sample completeness curve. **b** Rarefaction and extrapolation curves of species diversity base on the Hill numbers *q* = 0 (species richness) and *q* = 1(exponential of the Shannon index). The bands of each curve correspond to the confidence intervals calculated by 95%

It was confirmed that the sampling effort was adequate since high values (94.88%) of completeness of the sample were reached. For *q* = 0, it was estimated a species richness of 13 species, only one more species than observed (Fig. 4a). As for the exponential of the Shannon index (*q* = 1), an equivalent number of 10 equally common species was observed and 12 estimated (Fig. 4b).

### Local knowledge on stingless bees

The interviewees reported 15 different species of honey-producing bees with their local names (Table 2). Fourteen of them are stingless bees, and one is the Africanized honeybee (*Apis mellifera*), locally known as Abeja extranjera. Among the stingless bees, the most cited and valued species were Señorita which was mentioned by all interviewees, followed by Tancarillo and Negro. Other the species locally managed in the meliponaries were identified as *Tetragonisca angustula, Melipona rufiventris* and *Scaptotrigona sp*. As summarized in Table 2, local people identified bees based on morphological and behavioral characteristics, and for some species, they were also able to describe their nesting biology.

**Table 2:** Local knowledge on stingless bees at the municipality of Muyupampa and Monteagudo, Chuquisaca

Local name	Scientific name	Size	Predominant color	Behavior	Nesting place	Nest entrance	Mention index (%)
Señorita	*Tetragonisca angustula*	S	Yellow with black or brown	Docile	In hollow trunks, branches, or logs, underground, on the walls	Entrance cylindrical	100.0
Negro	*Scaptotrigona sp.*	M	Black	Aggressive	Mostly in hollow trunks or logs, some underground	Entrance cylindrical, bigger than from Señorita. When the entrance is big, bees are aggressive. When the entrance is small bees are docile	71.7
Tancarillo	*Melipona rufiventris*	L	Yellow/orange, with black stripes	Docile	In hollow trunks or logs	Entrance without big ornament, build with mud	45.0
Abeja Extranjera	*Apis mellifera*	L	Yellow and black	Aggressive	In hollow trunks or logs, underground	Entrance without ornament	41.7
Burro	Undefined	M	Black	Docile	In hollow trunks or logs, underground	Entrance cylindrical big and long	23.3
Sapito/Boca de sapo	*Plebeia sp.*	S	Black	Docile	In hollow trunks or logs	Entrance looks like a frog’s mouth	21.7
Burra	Undefined	M	Black	Docile	In hollow trunks or logs, underground	Entrance cylindrical and big	10.0
Señora	Undefined	M	Yellow	Undefined	In hollow trunks or logs	Entrance cylindrical	5.0
Chogñiri	Undefined	VS	Black	Docile	In hollow trunks or logs	Entrance cylindrical	5.0
Frontino	Undefined	S/M	Black, with a little white	Aggressive	In hollow trunks or logs	Entrance without ornament	3.3
Sañaro	Undefined	M/L	Yellow	Aggressive	In hollow trunks or logs	Entrance without ornament	3.3
Ilimchupa	Undefined	S	Black	Docile	Underground	Entrance without ornament	1.7
Hallpa negra	Undefined	M	Black	Aggressive	Underground	Entrance without ornament	1.7
Norita	*Paratrigona sp.*	S	Black, with white forehead	Docile	Underground	Entrance cylindrical	1.7
Chañar	Undefined	S	Black	Aggressive	In hollow trunks or logs	Entrance cylindrical	1.7

Local names are mostly in Spanish. Chogñiri and Hallpa Negra are in Quechua. Ilimchupa and Chañar are in Guarani

The well-known Señorita (*T. angustula*) was recognized by its slim body, small size, yellow–black colors (sometimes also described as dark brown), and very docile behavior. Local people know that this bee builds its nest in a variety of places, including hollow trunks, branches, or logs, underground or even on the wall or poles of the houses, and they build a small yellowish cylindrical cerumen entrance. Another well-known species (mentioned by 71.7% of the interviewees) is the totally black bee Negro (*Scaptotrigona* sp1), characterized by its medium size, and by the long cylindrical ornament, they build at the entrance of its nest at hollow trees or underground. According to most people, they have very aggressive behavior; however, some people described it as docile, which could be a confusion with another *Scaptotrigona* species. One person said that the bees were aggressive when the entrance was big, and the bees were docile when the entrance was small. Almost half of the local people (45%) mentioned the bee Tancarillo (*M. rufiventris*), distinguished by its large size, its yellow/orange color with black stripes, and its docile behavior. It was mentioned that this bee nests on hollow trees and logs and is hard to find since it builds small entrances. Two other black bees, Burro and Burra (cited by 23.3% and 10% of the interviewees, respectively) were described very similarly to the Negro, as docile, midsize, completely black, and nests with long cylindrical entrances built on hollow trees or underground. The bee known as Boca de sapo or Sapito was mentioned by 21.7% of the interviewees and identified by its small size, black color, and docile behavior. They nest in hollow trees and build around the entrance a characteristic ornament that reminds of a frog’s mouth. The other eight stingless bee species were cited by 5% or less of the interviewees. There was no significant difference neither in the influence of age (*p* = 0.698) or ethnicity (*p* = 0.554) on the number of species known by the local people, nor in the interaction between age and ethnicity on the number of known species (Additional file 1: Appendix 1 and Appendix 2).

When asked about the places where stingless bees were most commonly seen, 70% of the interviewees answered the forest with Señorita being the most common bee. These bees can also be seen in other places, such as the riverside, open fields, crops, or orchards, but not as often as in the forest. Nevertheless, many of the interviewees (43%) affirmed that in the last 5 years the population of bees had decreased and that this was principally because of the wildfires, climate change and pesticides, but also because the nests were withdrawn from the forest to be bred in boxes. On the other hand, another 39% of the interviewees said that the bee population had actually increased in the last 5 years mostly because of artificial breeding (Table 3). Despite these different perceptions, most of these local meliponiculturists (95%) agreed that bees generally benefit humans and the environment. Honey production was the most mentioned benefit that bees brought (mentioned by 62% of the interviewees) followed by politization (47%), propolis production (23%), and medicine (10%).

**Table 3:** Informants’ perception on the abundance and conservation status over the last 5 years of local stingless bees

Habitats where bees are more abundant	Informants’ perception (%)	Most abundant species	Informants’ perception (%)	Conservation status in the last 5 years	Informants’ perception (%)
Forest	70	Tancarillo	33	Increase	39
Crops	7	Señorita	48	Decrease	43
River side	8	Negro	18	Remains stable	5
Open field	8	Sapito	2	Do not know	13
Other	13	Other	13	–	–

^*^Some percentages add up more than 100% because the interviewees gave more than one answer in some cases

### Management techniques

Most interviewees had between 1 and 4 training courses held mainly through the PASOS Foundation. The most attended topics were the following: (1) management of modern hives, attended by 48 of the interviewed women. This course focused on the breeding of bees: the parts of a rational hive (boxes), artificial feeding to strengthen the colonies, divisions, and how to move wild colonies to rational hives. (2) Harvest of honey and other bee products, attended by 41 of the interviewed women. This course aimed to show how to harvest and later to preserve the honey and other hive products, taking into account sanitary measures. (3) Main characteristics of stingless bees, attended by 33 of the interviewed women. This course explained most of the characteristic traits of these bees, such as their social behavior and caste system, reproduction, what they feed on, and how honey is produced.

The most frequently managed stingless bee of this region is Señorita which is being bred by all the interviewees (100%), followed by Negros bred by 20% of the interviewees, Tancarillos 14%, and Sapitos 8.5%. Every meliponiculturist owns between 1 and 25 hives of Señorita, between 1 and 10 hives of Negro, and between 1 and 3 hives of some of the other stingless bee species. On the other hand, *A. mellifera* is being managed by 68% of the interviewees. Adding all the hives reported by the interviewees, there are in total 702 hives of honeybees, 617 hives of Señorita, 38 hives of Negro, and 27 hives of Tancarilo. A strong significant positive correlation (*p* = 0.0072, *r* = 0.881) was found between the percentage of people that own hives of a bee species and the mentioned index of such species, as shown in Table 2.

Some meliponiculturists (22%) obtained their hives by buying them, probably from other local meliponiculturists, while others (6%) got them through traps, dividing them, or as a donation. But most of the stingless bee hives (93%) owned by the interviewees originated from wild hives that were removed from their natural environment in the forest and placed inside wooden boxes. To remove these hives, around 33% of the times the trees or branches supporting the nest were cut off; in the other cases, the hives could just be removed since it was inside a dead log, or it was easier to remove them without cutting off the tree or branch. The survival rate of the hives that underwent these transfer processes was very high according to 55% of the interviewees, but 30% stated that some or most of the hives did not survive the transfer to the box. Once the hives were in the boxes, they were kept mostly around the house or in the meliponaries, where they were taken care and managed in different ways. In 88% of cases, hives hosted some pests in the last 5 years, mostly Phoridae flies, and attacks by other bees or ants. Most interviewees said they gave their hives artificial food (a mixture of water and sugar) to strengthen them when they looked weak, but less than 30% said they took preventive measures against ants, cleaning out the spiders’ nets or others. Regarding the artificial division of the colonies, 62% of the interviewees reported that they practiced this normally once a year, and most of them were successful.

Meliponiculturist who succeeded in artificial division of hives had taken a significantly higher number of training courses compared with those who did not perform artificial divisions (*p* = 0.0423). Similarly, those meliponiculturists who gave supplementary food to the hives, had taken a significantly higher number of training courses compared with those who did not perform this practice (*p* = 0.0095). On the other hand, there was no significant difference between the number training courses taken and the success or failure on racking (*p* = 0.723) or in the presence or absence of practices to fight pests (*p* = 0.340) (Table 4).

**Table 4:** Results for the generalized linear models for the influence of the number of training courses on the use and success of management techniques

Subject classes	Estimate	Standard error	*z* value	Pr (>|*z*|)
(A) influence of training courses on artificial divisions				
Success in division versus failure in division	− 0.183	0.194	− 0.942	0.35
Success in division versus no division	− 0.29	0.139	− 2.078	0.042
(B) influence of training courses on artificial feeding				
Supplementary feeding	0.451	0.168	2.684	0.009
(C) influence of training courses on racking the hives				
Success on racking	0.068	0.19	0.357	0.723
(D) influence of training courses on plague management				
Practice of pest management	− 0.117	0.121	− 0.964	0.34

The explanatory variables tested were: (A) colony division (success, failure, and no division), (B) supplementary feeding (yes and no), (C) racking of wild hives into boxes (success and failure), and (D) practice of pest management (yes and no)

### Stingless bees’ products, productivity, and commercialization

When questioned about the harvesting of the honey, 17% of interviewees said they did not harvest their hives, at least not yet, and the rest said they usually did it once a year. Honey production of the colonies of Señorita varied between 0.5 and 1 L per colony per year, and rarely more than 2 L. In contrast, the colonies of Tancarillo and Negro produced a larger volume of honey, yielding between 1 and 4 L per colony; however, these species are rarely bred. Besides honey, most interviewees also harvested pollen, propolis, and cerumen in lesser amounts. This activity was mostly done in the company of a son or a daughter.

In the communities, honey, pollen, propolis, and cerumen are used but in very different proportions. Honey was the most used product from the hive. Over 90% of the meliponiculturists harvested honey from their hives, most of them at least once a year, in some cases two (26%) or even three (6%) times a year. It is valued for its medical uses, specially to treat eye diseases and common cold, as well as food and as a source of income since it is a very valued product in the market. Despite this fact, just 38% of the meliponiculturists produced enough honey to sell it, approximately half was sold to the public, and the other half was sold in bulk to local NGOs at a price of around USD 21 per liter. This price is four to five times higher than honey sold in bulk from *A. mellifera* which can be sold for USD 4 to 5 [50]. The rest consumed the honey themselves as food or as medicine. As mentioned previously, Señorita is the most managed bee in this area, although it normally produces between 500 mL and 1 L, whereas Negro or Tancarillo produce on average 4 L and 2.3 L, respectively. The second most harvested product was pollen, locally called “pan de abeja,” being harvested by 35 women (59%). Pollen is mostly used as food, but some women also have medicinal use for it as energizer or nutritional supplement mostly for kids with anemia. Pollen is eaten pure, mixed with banana or with honey. It is rarely sold to the public, generally with a price of USD 15 per kg. Propolis was harvested by 42% of the interviewed women, and they used it equally as medicine or for sale. It was sold principally to the public with a very wide range of price oscillating between USD 3 and 12 per 100 mL. Cerumen was collected only by 31% of women. It was mostly purified and used to strengthen other hives. Three interviewees said they used the cerumen as food; one said she used it as medicine, and just one sold it at USD 22 per kg. Finally, the brood cells were the least harvested and used product of the hive. Just two interviewees (3%) mentioned this medicinal/traditional use; by eating the brood cells, it is believed that it enhances fertility.

### The use of honey and its medicinal properties

There is a consensus among the interviewees that the honey produced by Señorita is the most beneficial and the one with more medicinal properties, being used for treating several diseases. According to the frequency of citations, the main therapeutic use was for respiratory system treatments. Treatment of eye problems was cited almost as frequently (18 times), but in this case, honey was not consumed/eaten; instead, a drop or two are released directly on the affected eye. Ten interviewees also mentioned that consuming honey helped with digestive problems, and 9 said it strengthened the body and the immune system. In just a few cases (2), it was mentioned that honey could cure skin damages.

Regarding the uses of the honey produced by the other bees, in three different occasions it was informed that the honey produced by Burro and Burra was used to increase fertility, and two women mentioned that the honey produced by Negro was also helpful to treat digestive and respiratory problems.

The interviewees also shared some recipes and treatments they used to treat diseases (Table 5). Although some of the secondary ingredients varied, mostly there was a consensus on which ingredients to use for certain illnesses and how to prepare them.

**Table 5:** Recipes and treatments based on honey and other products of the hive

Affection/illness	Ingredients	Preparation
Common cold, throat pain	Honey, propolis, lemon, chamomile, or tea	Mix honey, lemon, and a few drops of propolis in chamomille or black tea. Drink hot once or twice per day
Covid 19, severe colds	Honey, propol, garlic, onion	Mix 10 teeth of garlic, 10 lemons, half cup of honey and a small onion. Allow to ferment for 5 days. Drink a small cup in the morning on an empty stomach
Gastritis	Honey and pollen optional	Mix and drink in the morning and afternoon on an empty stomach
Ophthalmological conditions	Pure honey	One drop of pure honey in the eyes for 15–30 days

### Relative cultural importance

The interviewees reported 30 different specific uses (#U) for the products of five stingless bee species: Señorita (*T. angustula*), Negro *(Scaptotrigona* sp.), Tancarillo *(M. rufiventris*), Sapito (*Plebeia* sp.), and Burro/Burra (*Scaptotrigona* sp.). These specific uses were grouped into 4 categories (#C): commercialization, food, medicine, and others (Additional file 1: Appendix 3). Based on the RCI index*, T. angustula* was the most culturally important bee in the region, followed by *Scaptotrigona* sp. (Table 6). There was a significative positive relationship between the percentage of people that managed a bee species and the relative cultural importance index (*r* = 0.875, *p* = 0.0044).

**Table 6:** RCI relative cultural importance index

Bee species	Relative cultural importance				
	#C	#U	C	U	RCI
Señorita	4	29	1	0.97	98.33
Negro	3	7	0.75	0.23	49.17
Tancarillo	2	2	0.5	0.07	28.33
Sapito	2	2	0.5	0.07	28.33
Burro	1	1	0.25	0.03	14.17
Burra	1	1	0.25	0.03	14.17

*#C* number of categories of use, *#U* number of specific uses, *C* proportion of the total number of categories of use, *U* proportion of th*e total number of specific uses*

## Discussion

### Knowledge on bee diversity and the problematic of local names

Considering the number of species collected during this study, the number of local names mention in the interviews revealed an overall wide traditional knowledge among the meliponiculturists of the Chaco region. Twelve morphospecies of stingless bees were collected; however, 15 different local names were mentioned in the interviews, together with some characteristics of the species. It is possible that some species were not collected during this study since some bee species may not be effectively attracted to the pan traps or they are infrequent and difficult to collect with a net [54]. Also, it has been proved on several occasions that local knowledge can provide useful information about the ecological and natural history of stingless bees helping to better assess them and even recognize new species [52, 55, 56]. On the other hand, it is also possible that more than one name mentioned by the interviewees correspond to only one species since the people that were interviewed belong to different ethnic groups which may have different names for the bees. Reyes-González et al. [26] documented in Michoacan, Mexico, in three different geographical zones but within the same state, up to five different local names for the same species. In Bolivia, *Lestrimelitta limao* for example, is called Limoncillo among the Ayoreos due to its characteristic lemon-like smell, but is called Pichi de burro by the Guaranies because of its large nest entrance [16, 57]. In future works, it would be interesting to deepen this knowledge with more extensive workshops with meliponiculturists as well as knowledge holders, analyzing their classification according to linguistic, cognitive, and socio-ecological meanings [58].

### Traditional knowledge

Most of the participants in the study did not consider themselves to belong to a specific ethnic group, resulting in an underrepresentation in the analysis carried out to identify an influence of ethnicity on traditional knowledge. Nevertheless, this reflects what is actually happening in most of the communities from Villa Vaca Guzmán, where most people did not feel identified with an ethnic group, probably because of common processes of globalization due to proximity to larger cities and the integration of rural populations to the market economy [59]. In this sense, and based on our results, it appears that traditional knowledge on bees is not significantly different between ethnic groups involved in meliponiculture. Although processes such as globalization potentially result in the loss of traditional knowledge [59], traditional and cultural knowledge is dynamic and in many cases these processes lead to changes and transformation rather than loss [60, 61]. These changes can mean that indeed some traditional knowledge is lost, but they may also lead to the uptake of new knowledge and, when many ethnic groups live together, also to the homogenization of knowledge [62].

### The influence of formal education

The influence of the meliponiculture courses given by the NGOs have a significant impact in the current knowledge, management and perception of meliponiculturists. This has also been observed by Zocchi et al. [59] who showed how the influence of external actors can lead to a hybridization of the traditional knowledge and modern apiculture, refining local practices while increasing production. Similarly, Jaffe et al. [63] highlight how the influence of modern practices have a positive effect on the management of hives as well as of the forest.

### Influence in the traditional knowledge

Contrary to what was expected, there was no significant difference between ages regarding the knowledge about stingless bees. This could be because of the initiatives from the local NGOs to promote meliponiculture. This initiative helps indirectly to homogenize the knowledge of younger and older people on one hand through workshops (in this case is mostly formal knowledge). On the other hand, these workshops may serve as platforms for the exchange of knowledge between the participants. As Jaffé et al. [63] identified in their study, beekeeper networks were fundamental to better developing the activity through knowledge exchange, recognizing that more experience meliponiculturists are a source of knowledge and promoters of meliponiculture.

### Influence in management techniques

The workshops given by the NGOs also influence the management of the hives, since they give the beneficiaries knowledge of new technological and management techniques. Meliponiculturists who have attended many workshops (three or four) were more likely to use some of these techniques, such as supplementary feeding the hives or doing artificial divisions. This was also observed by Jaffé et al. [63] and by Escareño et al. [64], who highlight the importance of acquiring these management skills to make meliponiculture a sustainable practice and since, on the one hand, multiplying colonies through artificial divisions is not easy, while supplementary feeding is an essential part of bee management influencing colony growth, performance and survival. Also, Contreras et al. [31] affirm that new advances in management has “led to a new flourishing of meliponiculture.”

Although there was not a significant difference in the success or failure of the divisions regarding the number of workshops that women assisted, there was a greater success rate in comparison with communities that only used traditional methods to divide the hives. Pat-Fernandes et al. [65] documented a success rate of only 20% from the divisions made by the indigenous people from communities in Campeche, Mexico; similarly, Villanueva-Gutierrez et al. [29, 66] documented a success rate of only 17% in communities within the Zona Maya of Quintana Roo. These figures may be compared to the 47% success rate of the women in Muyupampa.

### Influence in the perception and relative cultural importance

In this study, 30 different specific uses for the bee products were recorded, from which 29 were attributed (but not exclusively) to *T. angustula,* making it the most versatile species, and so the most culturally important species. Similarly, Kujawska et al. [67] reported that honey from five different bee species in Argentina were used to treat 30 affections, and honey was consumed pure or prepared with other plant ingredients. Also in Argentina, Geisa and Hilgert [68] identified 17 medical uses for the honey of *Plebeia molesta*. Other studies documented fewer specific uses for the honey and other products; for example, Gonzalez et al. [52] reported nine specific uses for *Scaptotrigona hellwegeri* in Mexico, and Bhatta et al. [53] who reported 18 specific uses for *Tetragonula iridipennis* in Nepal. Consistently the most treated affection with stingless bee honey were respiratory problems.

The meliponiculture workshops focus mostly on one species, *T. angustula,* and this incentive to work mainly with this one species can also bias the perception of the meliponiculturists and shift the cultural importance to this species. For example, when asked about the most abundant bees, most agree that the most abundant was Señorita, followed by other commonly bred species. However, the most collected species was *Plebeia* sp, which was only mentioned by 2% of the interviewees as an abundant species. Since the women are more interested in the species they manage, they probably pay more attention to these bees, or search specifically for them when walking through the forest, so they perceive that they are more abundant. Additionally, Sapito had a relatively high mention index (21.7%), and, for the relative cultural importance index (28.33), it is the third most versatile species. According to Gonzalez et al. [52], more abundant species are more versatile since people have more opportunities to work with them, and to experiment and attribute uses to them. Probably Sapito was formerly often managed, explaining it’s relatively high mention index and RCI index, but with the incentives of the NGOs, Señoritas have become the most managed species, and the traditional knowledge of breeding other species can fade off. This is why although the influence of formal knowledge have great advantages, it is important to emphasize that these initiatives have to take into account the local biodiversity to strengthen the breeding and conservation of more than one species. It is also necessary that they have an understanding of the local traditional knowledge to find endogenous elements that can be mixed with modern techniques.

### Stingless bee products, productivity, and commercialization

A proper management is also necessary to maintain a healthy environment for the bees, avoiding unnecessary deforestation [63]. In turn, the conservation of bee habitats is of utmost importance to assure a good productivity in the meliponaries. If the availability of adequate food sources decreases, creating an imbalance between the number of bees and their food resources and thus surpassing the carrying capacity of the forest, the bee colonies would produce less honey, and it would have probably lower quality [69, 70]. According to our results, the mean production of Señorita is around 500 ml to 1 L, and in some cases even up to 2 L per colony per year. This is the best productivity expected for this bee species [71, 72], and one of the key factors to achieve this is the quality and quantity of the food source that the bees have access to [69]. The production of honey is not only used for self-consuming, but it is also sold and has become an alternative for economic incomes. In the cities, the honey of Señorita is sold mostly as medicine and therefore in small containers of 20 or 30 mL, and it is sold for USD 2–3. This price is similar in other parts of Latin America; for example, in Costa Rica 10-mL containers cost USD 2–4 and 1-L containers up to USD 50 [72], and in Brazil 1 kg of *T. angustula* honey costs USD 20–30 [73]. The other products of the hive are mostly used for self-consumption.

## Conclusions

Our results showed a high diversity of native bees in the region that are recognized by the communities and represent an important income for the economy of meliponiculturists, especially women, as well as their families and communities. Participation in training that foster the use of modern meliponiculture methods shows better results with hive management and production. It is crucial that these initiatives have a broader perspective, not just focused on one species so that the communities will value cultural richness and biodiversity. On the other hand, it is important that small producers also develop formal competences for the commercialization and elaboration of derived products to promote sustainable development in the region.

## Supplementary Information

**Additional file 1. Appendix 1**. Results for the Generalized Linear Model for the influence of age and ethnicity on the number of species known be the local meliponiculturists. **Appendix 2**. Influence of age and ethnicity on the knowledge of stingless bees' species. **Appendix 3**. Categories of uses (#C), and specific uses (#U) of all the reported bee species (Common names) in the region of Monteagudo. Abbreviations: H = honey; Po = pollen; Pr= propolis; Ce= Cerumen.

## Data Availability

The data that support the findings of this study are available from the corresponding author upon request.
