# Supplementary material for: Stingless bees: uses and management by meliponiculturist women in the Chaco region of Bolivia
Source: J Ethnobiol Ethnomed. 2023 Jan 10;19:5. doi: 10.1186/s13002-022-00574-0 (PMC9830929; doi:10.1186/s13002-022-00574-0)
Supplement: Supplementary file 1 — Additional file 1. Appendix 1. Results for the Generalized Linear Model for the influence of age and ethnicity on the number of species known be the local meliponiculturists. Appendix 2. Influence of age and ethnicity on the knowledge of stingless bees' species. Appendix 3. Categories of uses (#C), and specific uses (#U) of all the reported bee species (Common names) in the region of Monteagudo. Abbreviations: H = honey; Po = pollen; Pr= propolis; Ce= Cerumen. [file 13002_2022_574_MOESM1_ESM.docx]

Additional file 1

Appendix 1. Results for the Generalized Linear Model for the influence of age and ethnicity on the number of species known be the local meliponiculturists.

| Difference in the influence of age and ethnicity on local people knowledge | | | | |
| --- | --- | --- | --- | --- |
| Subject Classes | Estimate | Standard Error | t value | Pr (>\|z\|) |
| Adult-Guarani vs Young-Guarani | 0.16 | 0.411 | 0.39 | 0.698 |
| Adult-Guarani vs Adult-Mestizo | -0.072 | 0.247 | -0.294 | 0.77 |
| Adult-Guarani vs Adult- Quechua | -0.245 | 0.411 | -0.596 | 0.554 |
| Adult-Guarani vs Young-Mestizo | -0.271 | 0.446 | -0.608 | 0.545 |
| Adult-Guarani vs Young-Quechua | -0.08 | 0.612 | -0.131 | 0.896 |


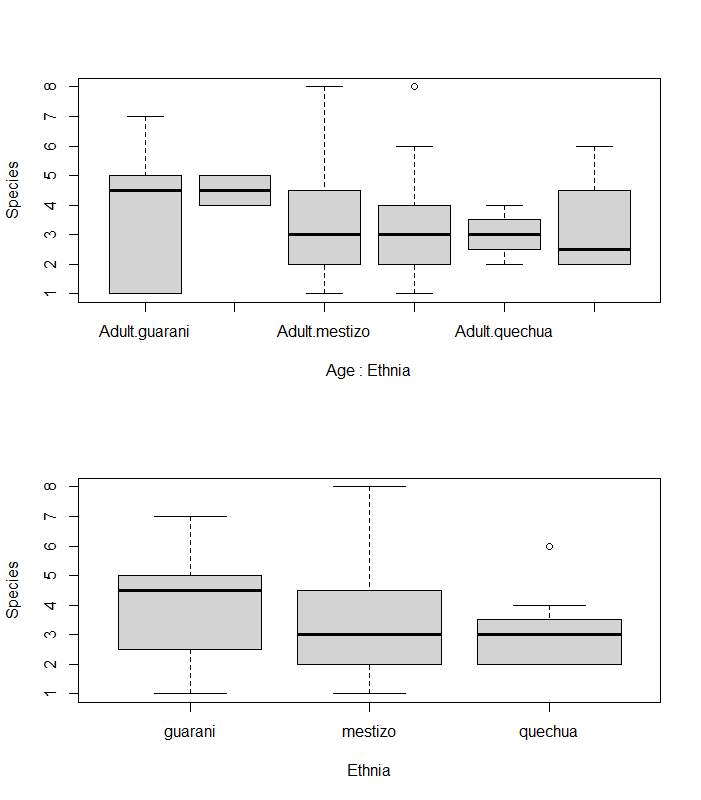


Appendix 2. Influence of age and ethnicity on the knowledge of stingless bees' species

Appendix 3. Categories of uses (#C), and specific uses (#U) of all the reported bee species (Common names) in the region of Monteagudo. Abbreviations: H = honey; Po = pollen; Pr= propolis; Ce= Cerumen.

|  |  | **Bee species** | | | | | | |
| --- | --- | --- | --- | --- | --- | --- | --- | --- |
| **Categories of use (#C)** | **Specific uses (#U)** | **Señorita** | **Negro** | **Tancarillo** | **Sapito** | **Burro** | **Burra** | **Apis** |
| **Comercialization** | Sell (H) | x | x | x | x | x | x | x |
|  | Sell (Pr) | x |  |  |  |  |  |  |
|  | Sell (Po) | x |  |  |  |  |  |  |
|  | Sell (Ce) | x |  |  |  |  |  |  |
|  | Barter (H) | x |  |  |  |  |  |  |
| **food** | Honey | x | x | x | x |  |  | x |
|  | Pollen | x | x |  |  |  |  |  |
|  | Propolis | x |  |  |  |  |  | x |
|  | Cerumen | x |  |  |  |  |  |  |
| **Medicine** | Eye problem (H) | x |  |  |  |  |  |  |
|  | External wounds (H) | x |  |  |  |  |  | x |
|  | External wounds (Pr) | x |  |  |  |  |  | x |
|  | Sprains (Ce) | x |  |  |  |  |  |  |
|  | Stomach problems (H) | x | x |  |  |  |  |  |
|  | Stomach problems (Pr) | x |  |  |  |  |  |  |
|  | Stomach problems (Po) | x |  |  |  |  |  |  |
|  | Respiratory problems (H) | x | x |  |  |  |  | x |
|  | Sore throat (H) | x |  |  |  |  |  | x |
|  | Sore throat (Pr) | x |  |  |  |  |  |  |
|  | Common cold (H) | x | x |  |  |  |  | x |
|  | Common cold (Pr) | x |  |  |  |  |  |  |
|  | Common cold (Po) | x | x |  |  |  |  |  |
|  | Anemia (Po) | x |  |  |  |  |  |  |
|  | Anemia (H) | x |  |  |  |  |  |  |
|  | Internal wounds (H) | x |  |  |  |  |  |  |
| **Others** | Fertility (H) |  |  |  |  | x | x |  |
|  | Energizing (H) | x |  |  |  |  |  |  |
|  | Energizing (Po) | x |  |  |  |  |  |  |
|  | gives appetite (Po) | x |  |  |  |  |  |  |
|  | to strengthen the hives (Ce) | x |  |  |  |  |  |  |
| Total #C=4 | Total #U=30 | #U=29 | #U= 7 | #U=2 | #U=2 | #U=2 | #U=2 | #U=8 |
